# Redox-Active Ferrocene Polymer for Electrode-Active Materials: Step-by-Step Synthesis on Gold Electrode Using Automatic Sequential Polymerization Equipment

**DOI:** 10.3390/polym15173517

**Published:** 2023-08-23

**Authors:** Hao-Xuan Guo, Yuriko Takemura, Daisuke Tange, Junichi Kurata, Hiroyuki Aota

**Affiliations:** 1 Department of Chemistry and Materials Engineering, Kansai University, Suita 564-8680, Osaka, Japan; toatiatua@gmail.com (Y.T.); k170289@kansai-u.ac.jp (D.T.); 2 Department of Mechanical Engineering, Kansai University, Suita 564-8680, Osaka, Japan; kurata@oram.co.jp

**Keywords:** redox capacitor, redox-active polymer, gold electrode

## Abstract

Redox-active polymers have garnered significant attention as promising materials for redox capacitors, which are energy-storage devices that rely on reversible redox reactions to store and deliver electrical energy. Our focus was on optimizing the electrochemical performance in the design and synthesis of redox-active polymer electrodes. In this study, a redox-active polymer was prepared through step-by-step synthesis on a gold electrode. To achieve this, we designed an automatic sequential polymerization equipment that minimizes human intervention and enables a stepwise polymerization reaction. The electrochemical properties of the polymer gold electrodes were investigated. The degree of polymerization of the polymer grown on the gold electrode can be controlled by adjusting the cycle of the sequential operation. As the number of cycles increases, the amount of accumulated charge increases proportionally, indicating the potential for enhanced electrochemical performance.

## 1. Introduction

Redox-active polymers have emerged as promising materials for redox capacitors, which are energy-storage devices that rely on reversible redox reactions to store and deliver electrical energy [1,2,3,4,5,6,7,8,9,10,11,12,13]. Redox-active polymers, which offer high energy density, serve as ideal electrode materials because of their high charge storage capacity, good cycling stability, and ability to undergo reversible redox reactions. These polymers typically comprise conjugated [3,4] or non-conjugated [5,6] main or side chains that incorporate redox-active moieties, such as transition metal complexes [7,8], organic radicals [9,10,11], or quinones [12,13], which are capable of undergoing reversible oxidation and reduction processes.

The design and synthesis of redox-active polymer electrodes are focused on optimizing their electrochemical performance. Several parameters, including redox-active units, polymer structure, molecular weight, and design of the molecular-level electrode surface, were carefully optimized to enhance the charge storage capacity, stability, and conductivity of the polymer electrode [14,15,16,17,18]. Emphasizing the design of the molecular-level electrode surface is crucial because it plays a vital role in optimizing polymer morphology and electrode configuration, thereby facilitating efficient charge transport and minimizing energy loss within the capacitor system. Although dip- and spin-coating methods are commonly employed for fabricating polymer electrodes, concerns have emerged regarding polymer orientation and electrode stability. However, these concerns can be effectively addressed by utilizing the Self-Assembled Monolayer (SAM) [19], which involves bonding gold and thiophene to prepare electrodes. SAM provides a solution for achieving a desirable polymer orientation and enhancing the bonding between the polymer and electrode [20,21,22,23].

Our primary focus was the electrode design. Our approach aims to achieve the sequential reaction of monomers on the electrode surface instead of simply attaching the polymer to the electrode. This approach offers several advantages, including the suppression of polydispersity and ease of controlling the degree of polymerization. By adjusting the degree of polymerization, precise control over the maximum charge storage capacity can be achieved. Additionally, this method allows for the incorporation of various functionalities into the molecule by modifying the aldehyde group.

Previously, we reported a novel pseudo-living polymerization technique involving 1-methylpyrrole (MePyr) and aldehydes. This technique allows for a step-by-step reaction starting from the terminal monomer and facilitates the synthesis of polymers with a precisely controlled structure [24,25]. In this study, our objective was to polymerize a redox-active polymer on a gold electrode using MePyr and ferrocenecarboxaldehyde (FcA). To achieve this, we designed an automatic sequential polymerization equipment that minimizes human intervention and enables a stepwise polymerization reaction. Herein, we demonstrate the synthesis of a redox-active polymer on a gold electrode using an automatic sequential polymerization approach. Adjusting the cycle of the sequential operation allows for precision control over the degree of polymerization of the polymer grown on the gold electrode. Furthermore, as the number of cycles increases, the amount of accumulated charge increases proportionally, indicating the potential for enhanced electrochemical performance.

## 2. Materials and Methods

### 2.1. Materials

1-Methylpyrrole (MePyr), and 3-thiophenemethanol were purchased from Tokyo Chemical Industry (Tokyo, Japan). Ferrocenecarboxaldehyde (FcA), *p*-toluenesulfonic acid monohydrate (*p*-TS), tetrabutylammonium hexafluorophosphate (TBuAPF_6_), ferrocene (Fc), tetrahydrofuran (THF), dimethyl sulfoxide (DMSO), and propylene carbonate were purchased from FU-JIFILM Wako Pure Chemical Corporation (Osaka, Japan). The MePyr monomer was purified via distillation prior to its use.

### 2.2. Measurements

Electrochemical measurements were conducted using a three-electrode system. The ALS660B electrochemical analyzer was employed for the measurements. ALS660B electrochemical analyzer, Pt working electrode, and Ag/AgCl reference electrode were purchased from BAS corporation (Tokyo, Japan).

### 2.3. Synthesis

#### 2.3.1. Automatic Sequential Polymerization Equipment

**Figure 1:**
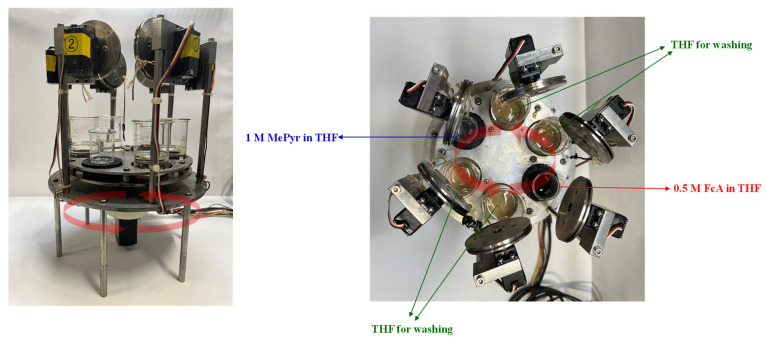
Image of automatic serialization equipment.

The automatic sequential polymerization equipment (Figure 1) used in this study was specifically designed by J. Kurata. This equipment was developed to enable precise and efficient step-by-step polymerization reactions on an electrode surface. By minimizing human intervention and integrating automated control, our equipment allows for the systematic synthesis of redox-active polymers with enhanced properties. In addition, the immersion and washing times in the experimental procedure could be adjusted according to the specific requirements of the synthesis. By modifying these parameters, we optimized the reaction conditions and controlled the growth and attachment of the redox-active polymer to the gold electrode.

#### 2.3.2. Preparation of Polymer Electrodes

The preparation of the gold electrode involved several steps. Firstly, a 1.0 cm × 2.0 cm Indium Tin Oxide (ITO) glass substrate was thoroughly cleaned using an ultrasonic cleaning process. The substrates were sequentially immersed in water, methanol, and acetone for 10 min each. Following the cleaning process, a thin film of gold (100 nm) was deposited onto the substrate using vacuum vapor deposition. Subsequently, the electrode was annealed to enhance the crystallinity of the gold surface and ensure flatness.

Preparation of polymer electrodes using automatic sequential polymerization equipment (Figure 1): To prepare Solution 0, 0.0086 g of 3-Thiophenemethanol, 0.122 g of MePyr, and 0.014 g of *p*-TS were dissolved in 0.3 g of THF. The resulting solution was allowed to react at 60 °C for 24 h. After the reaction, the solution was diluted with DMSO to obtain a 1 mM solution. The gold electrodes were then immersed in the solution for 24 h. After immersion, the gold electrodes were washed with DMSO and THF. To prepare Solution 1, FcA (0.750 mmol, 0.161 g), *p*-TS (0.610 mmol, 0.116 g), and THF (1.5 mL) were mixed. The gold electrode was placed in Solution 1 and allowed to react for 30 s, followed by washing with THF. To obtain Solution 2, MePyr (1.5 mmol, 0.122 g), *p*-TS (0.075 mmol, 0.014 g), and THF (1.5 mL) were mixed together. The gold electrode was placed in Solution 2 and allowed to react for 20 s. Alternating between Solution 1 and Solution 2, this process was repeated for 20, 40, and 80 cycles.

Important note: It is important to use freshly prepared MePyr, FcA and THF washing solutions for each set of 20 cycles when preparing modified gold electrodes.

## 3. Results and Discussion

**Figure 2:**
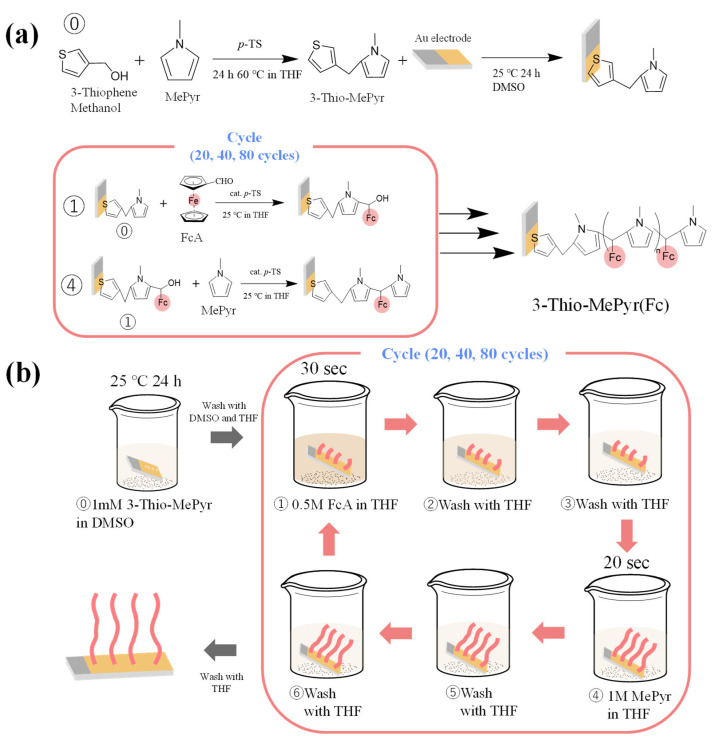
(**a**) Synthesis of redox-active polymer on a gold electrode using automatic sequential polymerization equipment; (**b**) preparation of polymer gold electrodes using automatic sequential polymerization equipment.

Redox-active polymers were synthesized via a step-by-step approach, involving the sequential synthesis of MePyr and FcA on gold electrodes using an automatic sequential polymerization apparatus. The redox behavior of ferrocene, which is characterized by the reversible transfer of electrons between its iron centers, makes it an excellent building block for the construction of electrochemically active materials [26]. Our goal is to systematically synthesize and characterize a redox-active ferrocene polymer on a gold electrode to explore its potential as an electrode material for various applications. To facilitate unidirectional growth of the polymer from the gold electrodes, 3-thiophenemethanol was chosen. The structures of the polymers are shown in Figure 2a. The step-by-step synthesis followed a specific order: Step (0) involved synthesizing the terminal groups of 3-thiophenemethanol and methylpyrrole, followed by binding these terminal groups to the gold electrodes using SAM. Step (1) entailed the reaction with FcA. Step (2) and Step (3) involved washing with THF. Step (4) entailed the reaction with MePyr. Step (5) and Step (6) included washing with THF. The sequence from Step (1) to Step (6) constituted one cycle (further details are provided in Section 2.3.2; a video demonstrating one cycle of the step-by-step synthesis process is included in Supplementary Materials). We performed three sets of syntheses with 20, 40, and 80 cycles. The preparation process for each set is illustrated in Figure 2b.

**Figure 3:**
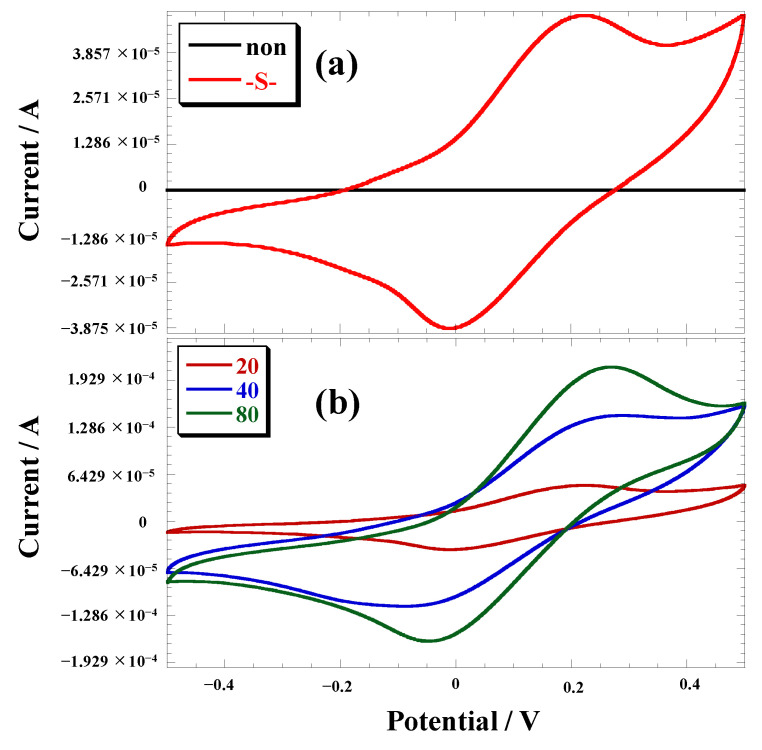
Cyclic voltammograms of polymer gold electrodes in 0.1 M TBuAPF_6_ propylene carbonate solution, (**a**) polymer synthesis on the gold electrode with (-S-) and without (non) thiophene, (**b**) polymer gold electrodes of syntheses with 20, 40, and 80 cycles, reference electrode: Ag/Ag^+^, at a sweep rate of 50 mV/s.

The electrochemical measurements were conducted using a three-electrode system, with the polymer gold electrode serving as the working electrode, Ag/AgCl as the reference electrode, and a Pt wire as the counter electrode. Cyclic voltammograms of the polymer gold electrodes were recorded in a 0.1 M TBuAPF_6_ propylene carbonate solution, and the results are presented in Figure 3 and Table 1. The redox waves originating from ferrocene were detected in all polymer gold electrodes. Conversely, sequential reactions carried out in the absence of thiophene did not lead to the synthesis of polymers on the gold surfaces. This finding suggests that the successful execution of sequential reactions on the gold electrode was facilitated by the SAM, as depicted in Figure 3a, highlighting the importance of incorporating thiophene in the process.

**Figure 4:**
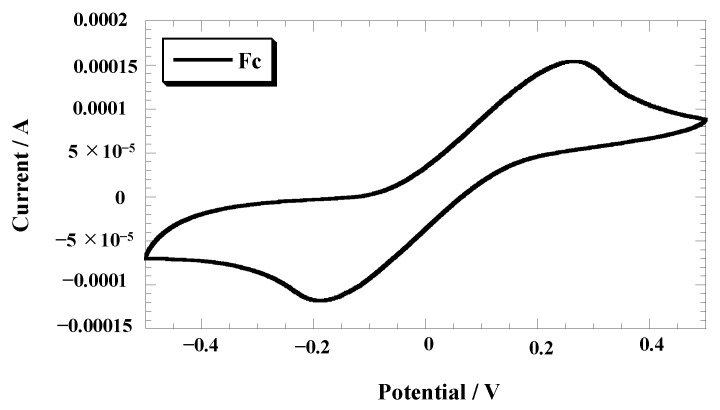
Cyclic voltammogram of Fc in 0.1 M TBuAPF_6_ propylene carbonate solution, reference electrode: Ag/Ag^+^ at a sweep rate of 50 mV/s.

The current value progressively increased with an increasing number of cycles, indicating gradual polymerization of the polymer from the electrode surface (Figure 3b). Notably, the width of the oxidation and reduction peaks in the redox wave of the polymer electrode was narrower than that of ferrocene in solution (Figure 4; CV measurement was performed on the ferrocene solution utilizing a gold electrode with the same dimensions of 1.0 cm × 1.0 cm as the gold electrode used for the polymerization process). This can be attributed to the binding of the ferrocene-based polymer to the electrode through the SAM, resulting in a more confined electrochemical environment. The cyclic voltammograms of the species adsorbed on electrodes typically exhibit sharp peaks. However, in this particular system, the redox wave of the adsorption system did not exhibit sharp peaks. This deviation from expected behavior can be explained by the redox reaction of the Fc groups in the polymer, which involves the incorporation of counterions that diffuse through the polymer to maintain electrical neutrality. It is likely that the current observed in the experiment was influenced by the diffusion of these counterions.

**Figure 5:**
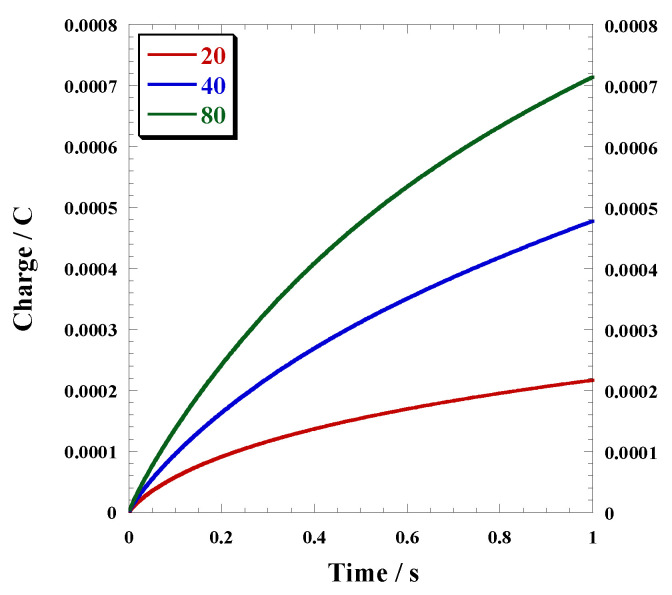
Chronocoulometry of polymer gold electrodes in 0.1 M TBuAPF_6_ propylene carbonate solution at 0.2 V taken for 1 s.

Figure 5 illustrates the electrode charge transfer for each of these polymer electrodes, as measured during the 1 s chronocoulometric measurements. The corresponding charges recorded at 1 s are listed in Table 1. The amount of charge accumulated on the polymer electrodes produced by different cycles was found to vary. In addition, consistent with the CV results, the amount of accumulated charge increased with the number of cycles. This observation strongly suggests that the polymer grows on the gold electrode as the number of cycles increases.

## 4. Conclusions

In conclusion, we have successfully demonstrated the synthesis of a redox-active polymer on a gold electrode using a step-by-step approach, employing an automatic sequential polymerization apparatus. By controlling the cycle of the sequential operation, we achieved precise control over the degree of polymerization of the polymer grown on the gold electrode. Furthermore, our observations revealed a direct correlation between the number of cycles and amount of accumulated charge, indicating the potential for further optimization and enhancement of the electrochemical properties of the polymer. These findings highlight the potential of redox-active polymers synthesized on gold electrodes for high-performance energy storage applications. Our ongoing research focuses on the development of redox capacitors with increased energy densities, achieved through improved sequential cycle numbers and electrode design.

## Figures and Tables

**Table 1:** Electrochemical properties of samples.

Sample	Redox Potential/V	Peak to Peak/V	Charges Recorded at 1 s/×10^−4^ C
20	0.11	0.231	2.17
40	0.107	0.361	4.78
80	0.111	0.313	7.14
Fc	0.035	0.430	-

## Data Availability

Not applicable.

## Supplementary Materials

The following supporting information can be downloaded at https://www.mdpi.com/article/10.3390/polym15173517/s1. Video S1: One cycle of the step-by-step synthesis process.
